# Immobilization of Growth Factors for Cell Therapy Manufacturing

**DOI:** 10.3389/fbioe.2020.00620

**Published:** 2020-06-19

**Authors:** Daniela Enriquez-Ochoa, Pedro Robles-Ovalle, Karla Mayolo-Deloisa, Marion E. G. Brunck

**Affiliations:** Tecnologico de Monterrey, School of Engineering and Science, FEMSA Biotechnology Center, Monterrey, Mexico

**Keywords:** cell therapy, growth factor, immobilization, stem cell factor, cost-of-goods, cell product manufacturing

## Abstract

Cell therapy products exhibit great therapeutic potential but come with a deterring price tag partly caused by their costly manufacturing processes. The development of strategies that lead to cost-effective cell production is key to expand the reach of cell therapies. Growth factors are critical culture media components required for the maintenance and differentiation of cells in culture and are widely employed in cell therapy manufacturing. However, they are expensive, and their common use in soluble form is often associated with decreased stability and bioactivity. Immobilization has emerged as a possible strategy to optimize growth factor use in cell culture. To date, several immobilization techniques have been reported for attaching growth factors onto a variety of biomaterials, but these have been focused on tissue engineering. This review briefly summarizes the current landscape of cell therapy manufacturing, before describing the types of chemistry that can be used to immobilize growth factors for cell culture. Emphasis is placed to identify strategies that could reduce growth factor usage and enhance bioactivity. Finally, we describe a case study for stem cell factor.

## Introduction

Growth factors (GFs) are signaling molecules that influence cell fate upon binding their cognate receptors. Their production is tightly regulated in healthy organisms, to specifically modulate cell physiology (Burgess, 2015). Once a GF binds its receptor, a signal transduction pathway initiates, leading to regulated gene expression, and the modulations in metabolism, protein synthesis, ion fluxes, and cytoskeleton organization responsible for the resulting phenotype at the cellular level (Lee et al., 2011). GFs may stimulate various biological pathways such as proliferation, differentiation, and survival (Cross and Dexter, 1991; Fortier et al., 2011). The effect of a GF is strongly influenced by its concentration, the nature of the target cell, the presence of co-stimuli, and the existence of said GF as a soluble molecule, or immobilized, embedded in the extracellular matrix (ECM) (Cross and Dexter, 1991; Pan et al., 2002). For example tumor necrosis factor α (TNF-α) exists either as a soluble or immobilized molecule *in vivo*, which was shown to define its role as selectively supporting or inhibiting tumor growth and survival in various tumor cell lines (Ardestani et al., 2013).

Due to the determinant role of GFs in cell fate, most culture protocols require their addition to culture media, to date mostly as soluble molecules, to produce cells of specific phenotypes for research or for therapeutic use. In this context, the use of GFs represents a major cost contributor in the production of cell therapies (Kirouac and Zandstra, 2008; Torres-Acosta et al., 2019). The recent clinical success of cell therapies, such as chimeric-antigen receptor (CAR) T cell therapies, combined with their costly production and the realization that GFs hugely contribute to this cost, evidenced a strong need to develop strategies to reduce GF usage (Torres-Acosta et al., 2019).

GFs used in soluble form exhibit accelerated depletion from culture media through low stability and bioactivity, which calls for constant replenishing. Immobilizing GFs avoids their internalization post-receptor binding, which may enhance or preserve biological activity (Chen et al., 1997; Ito et al., 2001). Immobilization may allow spatial- and time-controlled presentation to target cells, for example when bound to the surface of magnetic particles. This feature could additionally open a window of opportunity for reuse (Zandstra et al., 1997; Samorezov and Alsberg, 2015; Worrallo et al., 2017). Immobilization of GFs may lead to their use in overall smaller quantities in cell culture, effectively decreasing cell production costs. However, techniques used to immobilize GFs are varied and choosing the most appropriate chemistry for a particular protein under a specific use is not trivial.

To date, the majority of the literature has focused on the production of immobilized GF (iGF) for tissue engineering applications (Lee et al., 2011; Cabanas-Danés et al., 2014; Reed and Wu, 2014; Hajimiri et al., 2015; Wang et al., 2017; Atienza-Roca et al., 2018). Therefore, this review aims to discuss general approaches to immobilize GFs in the context of cell therapy manufacturing. While tissue engineering usually involves the culture of adherent cells, cell therapy production deals with single cell suspensions at very large scale, usually in bioreactors (van den Bos et al., 2014; Pigeau et al., 2018). Here, we summarize the clinical and economic relevance of cell therapies and describe pertinent cell therapy manufacture bioprocesses to evidence areas of opportunity for the use of iGF. Then we describe the chemistry of current relevant strategies used to immobilize GFs and discuss their selectivity, bioactivity and release. Finally, we present the case of stem cell factor (SCF), a relevant GF used in stem cell research.

## Cost of Cell Therapy Development and Manufacturing: Flipside of the Coin

In contrast to drugs and bioactive molecules, cells respond dynamically to stimuli and tailor their phenotype *in vivo*, which gives cell therapies a possible curative edge for medical conditions exhibiting poor response rates using conventional medicines (de Wilde et al., 2016a). Encouraging clinical trials launched multiple cell therapies as novel alternatives to treat life threatening conditions including neurodegenerative and autoimmune diseases, cancer, congenital diseases, and metabolic disorders (Mason et al., 2011; de Wilde et al., 2016b). Between 2004 and 2014, European countries have welcomed testing of 198 cell and gene-based therapies in clinical trials (de Wilde et al., 2016a). The significant increase in number of clinical trials of cell-based therapies in recent years highlight the relevance of these treatments in the future of global health care.

The first *ex vivo* stem cell-based therapy to receive regulatory approval, Strimvelis, treats the rare congenital disease adenosine deaminase severe combined immunodeficiency (ADA-SCID) (Stirnadel-Farrant et al., 2018). Strimvelis is based on the modification of autologous CD34^+^ cells through *ex vivo* retroviral transduction, to express functional adenosine deaminase. Modified cells are infused back into the patient as a one-time injection of ≥ 4 million CD34^+^ cells/kg, homing to the bone marrow and repopulating healthy blood cells, in theory for the lifetime of the patient (EMA-European Medicines Agency, 2016). This therapy has demonstrated a 100% survival rate up to 7 years post-injection (EMA-European Medicines Agency 2016). In 2018, Strimvelis treatment cost was €594,000 (Stirnadel-Farrant et al., 2018).

Another example of a current FDA and EMA-approved therapy is Sipuleucel-T. This therapy is aimed to treat castration-resistant prostate cancer. Sipuleucel-T is produced by culturing the patient's own antigen presenting cells (APCs) such as monocytes and others, together with PA2024, a recombinant protein combining the antigen prostatic acid phosphatase to GF granulocyte-macrophage colony stimulating factor (GM-CSF). Exposing APCs to this combination of GF/antigen results in cell activation against cancer antigens, prompting a targeted anti-cancer response in the patient (Pieczonka et al., 2015). In 2011, Sipuleucel-T was commercialized by Dendreon (under the name Provenge), with a dose cost of US$169,206 (Shukla et al., 2019).

While APCs re-educate the immune system of the patient *in vivo* as seen with Sipuleucel-T, it is now possible to engineer *ex vivo* a patient's T lymphocytes to readily recognize and eliminate cancer cells. Tisangenlecleucel (Kymriah) is an FDA approved therapy based on chimeric antigen receptors (CAR) recombinantly expressed within a patient's own T lymphocytes cell membranes. These engineered CAR-T cells have shown success in treating acute lymphoblastic leukemia (ALL) in pediatric patients by targeting the CD19 antigen expressed by malignant B cells (Vairy et al., 2018). Tisangenlecleucel is manufactured by viral transduction of CD3^+^ lymphocytes enriched post-leukapheresis. Clinical trials evidenced 81% of patients in remission at 3 months follow up, from which 60% showed complete remission (Maude et al., 2018). However, the current therapy costs US$475,000 (Herper, 2018). A similar CAR T-cell therapy, Axicabtagene ciloleucel commercialized by Kite Pharma under the name Yescarta also targets CD19 together with a CD28 co-stimulation (Jain et al., 2018). Yescarta is produced using retroviral transduction and specifically targets non-pediatric patients with diffuse large B-cell lymphoma and non-Hodgkin lymphoma (EMA-European Medicines Agency, 2014; Food Drug Administration, 2017). The treatment with Yescarta increased 9.5 years life expectancy, vs. 2.6 years using conventional chemotherapy treatments. In 2018, Yescarta costed US$522,921 per treatment (Roth et al., 2018).

Despite the superior curative potential of cell therapies, their availability is strongly limited by their price tag. Only 10% of therapies currently in Phase I clinical trial will reach stage 4 (Heathman et al., 2015). In addition to safety and efficacy considerations for pursuing the implementation of a specific cell therapy, high production costs is a known deterrent for manufacturers (Heathman et al., 2015). Technoeconomic and cost-benefit analyses have emerged to determine whether these therapies are rationally implementable. The manufacturing process comprises ~90% of the total investment destined to develop a novel cell therapy (Vormittag et al., 2018). In both autologous and allogenic cell therapy manufacturing, the process comprises all steps post cell-sourcing, including washes, cell activation and proliferation, final product formulation, and quality controls (Vormittag et al., 2018). Amongst the factors that contribute to these manufacture costs, the purchase of materials necessary for cell culture, such as culture medium and supplements, are listed as strong cost contributors (Lipsitz et al., 2017; Torres-Acosta et al., 2019). For example, in the context of induced pluripotent stem cells which are a promising player in the cell therapy field, GFs constitute the majority of essential components promoting cell growth, with 4 out of 6 components of the “essential medium” E8 being GFs (Chen et al., 2011). Efforts are needed to optimize manufacturing processes in order to reduce cost of goods and boost accessibility to patients. One approach is the development and implementation of automation, to reduce labor costs (Heathman et al., 2015). Another alternative is the immobilization of GFs. As immobilization improves GF stability and prevents degradation, immobilized GFs (iGFs) may lead to their use in decreased quantities in comparison with cultures using their soluble counterpart (Worrallo et al., 2017).

Optimizing cell manufacturing is essential to the commercial success of cell therapies. Immobilizing GFs promises to significantly reduce GF usage and consequently, manufacturing costs (Lotz et al., 2013; Worrallo et al., 2017). In addition to economic saving, GF immobilization may induce enhanced signaling, evidenced by increased cell bioactivity (Kitajima et al., 2007; Boucher et al., 2010; Yang et al., 2012; Budiraharjo et al., 2013; Lotz et al., 2013; Kumorek et al., 2015). While to the best of our knowledge GF immobilization is not currently used to produce cell therapies, the immobilization of other media components has been implemented, providing a proof-of-concept for feasibility and implementation. Dynabeads are magnetic polymer particles which surface is covered with covalently immobilized antibodies. In clinically implemented protocols, Dynabeads are used as a culture media supplement to expand T cells through CD3 and CD28 antibody-mediated activation (Neurauter et al., 2007). Implementing cost-effective technologies that contribute to enhance and simplify production processes will become critical for cell therapy manufacturing at a relatively low cost (Wang and Rivière, 2016).

## Methods for GFs Immobilization

Current protocols for cell production involve frequent feeding with soluble GFs to control cell proliferation and phenotype. The use of iGFs is an attractive approach for cell production processes, as it offers various advantages. The presentation of GFs in an immobilized form allows receptor binding and activation at the plasma membrane, but prevents GF internalization and intracellular recycling, resulting in sustained cell stimulation (Chen et al., 1997; Ichinose et al., 2006; Rodrigues et al., 2013; Kim et al., 2016b). Of note, some physiological responses are positively regulated through receptor-ligand endocytosis. In such instances where endocytosis is necessary for the desired phenotype to be achieved, soluble GFs should be used (Ceresa, 2011). GF immobilization also improves GF stability, mimics a physiological ECM-bound presentation of ligands often occurring *in vivo*, and enables GF reuse without loss of bioactivity (Mizumachi and Ijima, 2013; Mao et al., 2017; Wang et al., 2020). Production systems incorporating iGFs have been developed for cells cultured in adherence and in suspension with the overall aim to improve the efficiency of culture processes (Rahman et al., 2010; Yang et al., 2012; Lotz et al., 2013; Mao et al., 2017; Worrallo et al., 2017). Usually adherent cells are cultured on surfaces incorporating immobilized GFs, and because of the relative ease of GF immobilization in these conditions, the majority of reports detail such protocols. More recently, cells growing in suspension have been cultured in contact with iGF, using a variety of approaches. For instance, GFs may be immobilized onto magnetic beads, or encapsulated, prior to their addition to the culture media (Lotz et al., 2013; Worrallo et al., 2017). Of note, adherent cells can be cultured in suspension through encapsulation within GF immobilized matrices (Mao et al., 2017). This is highly relevant for cell production processes as culturing cells in suspension increases real estate available in a defined culture vessel. Producing cells in suspension with the incorporation of iGF is an attractive additional optimization over current processes, which we propose to discuss in this review.

Two approaches can be used to immobilize GFs into surfaces: chemical and physical interactions. The former relies on attaching GFs through covalent or non-covalent bonds directly to the substrate surface or to molecules that are used as linkers between the immobilizing surface and the GF. The latter approach involves the entrapment or adsorption of GFs into a substrate, allowing a diffusion-based release. The nature of cells being produced dictates GF requirements, and a single GF or a combination of various GF may be necessary to produce the desired cell phenotype and quantity. Most available reports have focused on immobilizing a single GF to be investigated as soluble or immobilized in a complex medium. Nonetheless, the functionalization of different surfaces with multiple GFs has also been implemented using both chemical and physical approaches (Stefonek-Puccinelli and Masters, 2008; Shah et al., 2011; Banks et al., 2014; Lequoy et al., 2016; Mao et al., 2017; Cheng et al., 2019). Importantly, the synergistic or antagonistic effect that co-immobilized GFs may have on bioactivity strongly depends on the nature of GFs and their concentrations in culture, so that no generalization can be made about the immobilization of more than a single GF (Stefonek-Puccinelli and Masters, 2008; Banks et al., 2014; Mao et al., 2017).

## Physical Immobilization of GFs

**Figure 1:**
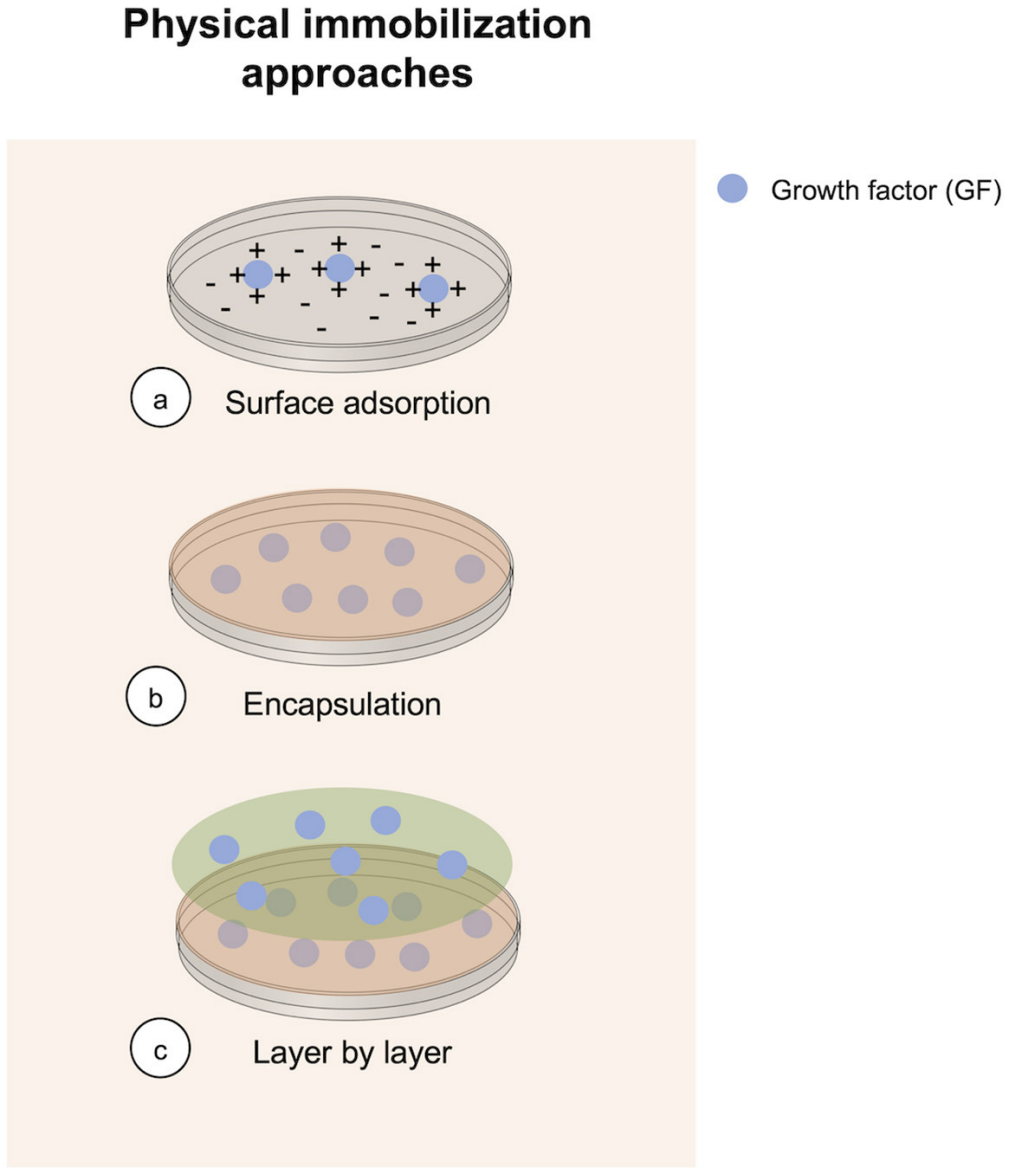
Overview of methods currently in use to perform physical GF immobilization.

Physical immobilization is technically the simplest method to immobilize a GF, and is commonly used for tissue engineering applications, specifically for bone regeneration purposes (Jensen et al., 2015; Ma et al., 2015; Nyberg et al., 2016; Hettiaratchi et al., 2017; Schumacher et al., 2017). It is commonly achieved by adding a determined number of GF into a polymer matrix before its gelatinization. There are three different approaches for performing GFs physical immobilization (Figure 1). Advantages of GF physical immobilization involve technical accessibility, low cost of reagents and preservation of bioactivity of the iGF. Furthermore, hydrogels used for GF immobilization by physical immobilization are suitable for cell scaffolding. However, poor spatial distribution and control over release are obtained using this method, explaining current efforts using different methods. Despite these drawbacks, physical immobilization remains a common method for achieving GF immobilization.

### Physical Encapsulation

Physical encapsulation is based on the immobilization of the GF into a matrix or scaffold. The interaction between the GF and the selected material for entrapment relies on hydrophobic, hydrophilic-hydrophilic, and electrostatic interactions (Lee et al., 2011). A wide variety of GFs have been immobilized using physical encapsulation for tissue engineering applications, including vascular endothelial growth factor (VEGF), GM-CSF, bone morphogenetic protein-2 (BMP-2) and insulin-like growth factor (IGF-1) (Rocha et al., 2008; Ali et al., 2009; Abbah et al., 2012; Hameed et al., 2019). Moreover, this technique enables the culture of non-adherent cells with iGFs, since GFs can be encapsulated and added to suspension culture systems, such as flasks and bioreactors.

To our knowledge, only one study has employed this particular immobilization method for the optimization of a cell production process (Lotz et al., 2013). The encapsulation of fibroblast growth factor-2 (FGF-2) into PLGA microspheres improved human embryonic stem cells (hESCs) phenotype and culture conditions. After 72 h of culture, the levels of FGF-2 released from the microspheres remained almost unchanged. Whereas, the levels of soluble FGF-2 decayed by 90%. In the cultures in which encapsulated FGF-2 was employed, an increase in expression of pluripotency markers was observed. Additionally, stem cell spontaneous differentiation was reduced significantly when cells were cultured with FGF-2 beads instead of soluble FGF-2. The controlled release of FGF-2 decreased the frequency of medium changes necessary to maintain hESCs culture from daily to biweekly feeding. These results demonstrate that the use of encapsulated FGF-2 is an effective method to lower consumable costs and labor required to maintain hESCs.

In another study, Lee et al. (2004) incorporated transforming growth factor-beta-1 (TGF-β1) loaded microspheres into porous chitosan scaffolds to support chondrogenesis. Encapsulated TGF-β1 promoted cartilage regeneration by significantly promoting chondrocyte adhesion, proliferation and glycosaminoglycan production compared to scaffolds without TGF-β1. Additionally, the chitosan scaffolds facilitated controlled release of TGF-β1, with an initial burst effect that stabilized after 3 days, and by day 7, 44.8% of the initial loading was released. These results indicate that encapsulation is an effective approach to control the release of GFs while maintaining their bioactivity.

Due to its biocompatibility and biomechanical properties, alginate is widely used for GF and cell encapsulation in tissue engineering and regenerative medicine applications (Hwang et al., 2009). Choi et al. (2010) developed a GF delivery system based on microcapsules made of poly(lactic-co-glycolic acid) (PLGA) and alginate. BMP-2 and dexamethasone were loaded into the microcapsules to facilitate osteogenic differentiation of rate bone marrow stromal cells (BMSCs). PLGA-Alginate microcapsules retained approximately 40% of the loaded GF after 30 days. BMSCs cultured with BMP-2-loaded microcapsules showed greater expression levels of osteogenic markers, such as collagen type I, osteopontin, ALP, and osteocalcin, compared to cells cultured with microcapsules lacking GF. Therefore, PLGA and alginate microcapsules are a delivery system of relevance, able to induce osteogenic differentiation in BMSCs when loaded with a GF. In another study, VEGF was encapsulated in calcium-alginate beads, where loading efficiency could reach 97% under optimized conditions. Further, a constant rate of 6 ng of GF/ml/day could be achieved and sustained for 14 days (Gu et al., 2004).

Nanoparticles have recently gained prominence as advanced drug delivery systems in the biotechnology and pharmaceutical industries. In the context of GF immobilization, nanoparticles can be loaded with GFs, facilitating controlled delivery (Wang et al., 2017). For instance, VEGF was loaded into dermatan sulfate sodium salt-poly-l-lysine (DS-PLL) and gum tragacanth-poly-l-lysine (GT-PLL) nanoparticles with loading efficiencies of 93.1 and 80.2%, respectively (Zandi et al., 2020). Nanoparticles -immobilized VEGF induced higher proliferation compared to soluble VEGF in HUVEC culture. Other GFs, including BMP-2, epidermal growth factor (EGF), FGF, insulin-like growth factor-1 (IGF-1) and TGF-β1, have been immobilized to nanoparticles to control spatial presentation in tissue engineering and regenerative medicine (Matsuo et al., 2003; Rajam et al., 2011; Ertan et al., 2013; Wang et al., 2016).

### Surface Adsorption

GF immobilization through surface adsorption is one of the simplest methods to generate functionalized matrices for biomedical purposes. It involves the use of a biocompatible matrix monolayer, with a GF attached to it. Depending on the GF and matrix material, the binding of the GF to the matrix will not only depend on electrostatic interactions, but also on hydrophobic ones (Luginbuehl et al., 2004). The adsorption of GF to the matrices may be modified by utilizing different types of materials with particular properties. The properties of materials and solutions used to immobilize the GF can be tuned to control adsorption (King and Krebsbach, 2012). Some of the disadvantages of this approach are low loading efficiency, poor control over release, and minimum spatial control (Midy et al., 1998; Budiraharjo et al., 2013; Wang et al., 2017). This approach has been utilized to immobilize different types of GFs, including BMP-2, FGF, and FGF-2 (Ziegler et al., 2008; Budiraharjo et al., 2013).

### Layer-by-Layer (LbL) Immobilization

In order to address poor control over release problem present in surface adsorption, LbL immobilization represents an alternative with improved spatial distribution and control over release the adsorbed GF. LbL is based in the incorporation of several layers of matrix with GF adsorbed on it. This immobilization strategy depends mainly on electrostatic interactions between GFs and oppositely charged electrolytes, although, hydrophobic interactions and hydrogen bonds are also present (Zhang et al., 2015; Wang et al., 2017). In addition to improving release control and spatial distribution, LbL is a simple and inexpensive technique. The efficiency of LbL does not depend on the GF size/shape, so multiple GFs can be attached by optimizing the architecture design (Gomes et al., 2015).

LbL is widely used for neural and cardiac tissue repair, bone regeneration, and wound healing approaches (Kulkarni et al., 2014; Lynam et al., 2015; Amano et al., 2016; Guduric et al., 2017; Liu et al., 2017; Mandapalli et al., 2017). As an example of this type of immobilization, Naves et al. (2016) created a LbL architecture based on poly(ethylene imine) (PEI) in combination with heparin and chitosan to immobilize acidic fibroblast growth factor (aFGF) and basic fibroblast growth factor (bFGF), respectively. The rate of GF release is inversely correlated to the number of layers in the matrix. Interestingly, increasing amounts of adsorbed GF lead to increased stability and lower release, and this phenotype has more impact in architectures of lesser layers. For both aFGF and bFGF a 6-bilayer architecture resulted significantly more stable than a 3-bilayer one. These results coincide with the release profile for bFGF for both 6 and 3 bilayer architecture, where after 14 days 0.4 ng/ml and 0.8 ng/ml of GF were released respectively. Both results indicate that 3 bilayer substrates can release GF faster than a 6 bilayer one, due to its lower stability. Regarding GF *in vitro* bioactivity; NIH 3T3 mouse fibroblast were used in culture. Although proliferation assays showed that both 3 and 6 bilayer substrates induced proliferation, it was the 3-bilayer architecture which significantly enhanced fibroblast proliferation, after the 14-day culture. Final cell count for aFGF 3 bilayer substrate was 1.8 times higher than the response achieved by the aFGF 6 bilayer substrate. These results can be easily explained due to the lower stability for 3 bilayers; hence this architecture will release a greater amount of GF over time, therefore enhancing cell proliferation. Additionally, long-term bioactivity for immobilized aFGF and bFGF was tested. Although cell proliferation was induced, it was not enhanced. Hence, bioactivity is maintained but not increased, this long-term activity can be attributed to the multilayer architecture protecting the GFs and preventing their denaturalization.

In another study, FGF-2 was immobilized into a 5 polyelectrolyte bilayer architecture, and used as substrate for cell culture (Ding et al., 2018). Polyelectrolytes used to adsorb FGF-2 were poly methacrylic acid (PMAA) and poly L-histidine hydrochloride (PLH), forming the resulting FGF-2(PMAA/ PHL)5 architecture. FGF-2 (PMAA/PHL)5 resulted in a constant release of the GF. In consequence fibroblast proliferation was slightly improved, when compared with GF added in a single dose. Kumorek et al. (2015) also utilized a LbL approach for immobilizing FGF-2, utilizing an albumin/heparin (Alb/Hep) 2 bilayer architecture. Before GF adsorption, the LbL was crosslinked covalently with glutaraldehyde, to stabilize the LbL substrate. To asses immobilized GF bioactivity, FGF-2 dependent cells were cultured in tissue culture plates coated with FGF-2 (Alb/Hep) 2 bilayer for 7 days. Results indicated that functionalized FGF-2 (Alb/Hep)2 surfaces enhanced proliferation and cell differentiation. Taken together, the results of the previously mentioned studies demonstrate that LbL is a simple and effective approach to achieve consistent release of GFs, opening the possibility to reduce costs and maintenance of adherent cells culture.

### Chemical Immobilization of GFs

#### Covalent Approaches for GFs Tethering

**Figure 2:**
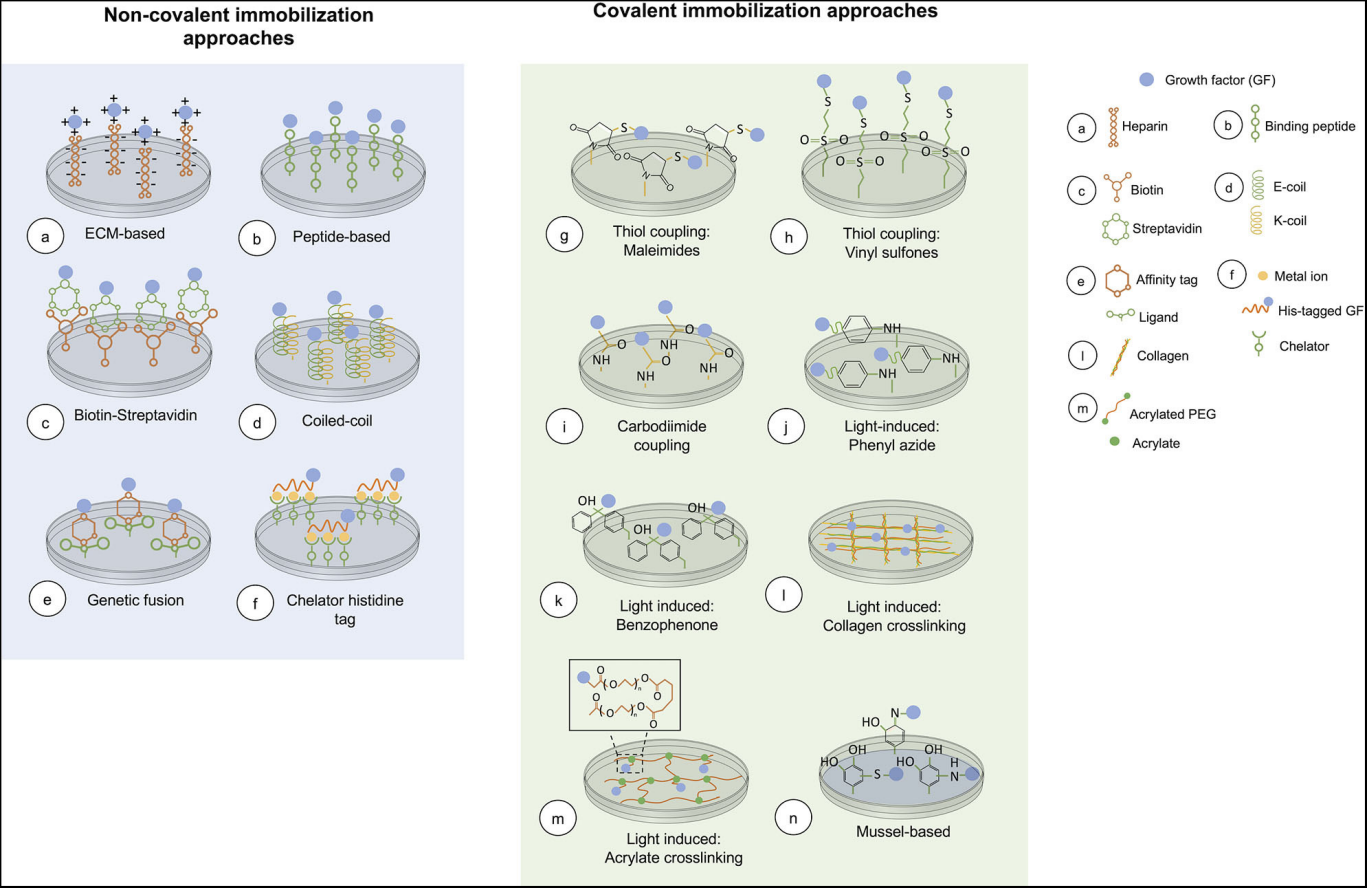
Overview of current chemical immobilization strategies. In covalent methods, acrylate crosslinking is representative of the conjugation of an acryloyl GF and PEG-diacrylate matrix. While, mussel-based immobilization shows the interaction between the GF and polydopamine coated on the surface.

Covalent immobilization of GFs to biomaterials usually engages functional groups in GFs for immobilizing them onto a surface. A major concern is that these functional groups may be near to the GF active site, affecting bioactivity when the GF is immobilized (Leipzig et al., 2010). In this type of immobilization GFs are attached onto the matrix strongly and irreversibly. Covalent binding of GFs to surfaces is required when these cannot be adsorbed onto a substrate surface, or when a gradual release from the substrate is necessary. An overview of covalent immobilization methods is presented in Figure 2.

##### Carbodiimide coupling immobilization

Carbodiimide coupling represents one of the most common methods for covalently attaching GFs onto substrate surfaces. Carbodiimides are cross-linking agents that mediate the reaction between amine groups and carboxylic acids to form amide linkages. Among the several carbodiimide reagents available, 1-ethyl3-(3-dimethylaminopropyl)carbodiimide hydrochloride (EDC) is the most used for bioconjugation processes. The conjugation reaction proceeds by the generation of an active *o*-acylisourea intermediate, which then reacts with a nucleophile such as a primary amine for amide bond formation. Unfortunately, *o*-acylisourea can hydrolyze in aqueous solutions, regenerate the carboxylic groups and fail to bind the target GF, decreasing the reaction efficiency. In order to prevent this reverse reaction, carbodiimides are mostly coupled with N-hydroxysuccinimide (NHS). NHS reacts with *o*-acylisourea and EDC to form a carboxyl-NHS ester, which is more stable in aqueous solutions, minimizing susceptibility to hydrolysis (Hermanson, 2008). In addition to linking GFs to the substrate, EDC may bind substrate molecules between themselves, resulting in a lower GF loading efficiency (Chiu et al., 2011). This problem can be attenuated by performing a step immobilization process: EDC activation of the substrate is performed first, followed by addition of GFs (Chiu et al., 2011).

Carbodiimide coupling may engage the amine groups in the GF lysine residues or N-terminus, as well as the carboxylic groups in the GF aspartate or glutamate residues or C-terminus. Due to this lack of specificity, the possible engagement of bioactive functional groups in bond formation may cause a GF to lose its bioactivity (Nakaji-Hirabayashi et al., 2007). Likewise, the presence of multiple functional groups, causes GF immobilization in a random orientation, which consequently affects epitope availability for the cognate cell receptors (Masters, 2011).

As an example, Psarra et al. (2015) reacted hepatocyte growth factor (HGF) and basic fibroblastic growth factor (bFGF) amine groups with the carboxylic groups on poly(acrylic) acid (PAA) brushes to achieve immobilization through carbodiimide coupling (Psarra et al., 2015). The effect of GF functionalized PAA brushes was investigated on different cell lines. In a human hepatoma cell line (HepG2), covalently immobilized HGF demonstrated higher bioactivity than the soluble GF with a concentration 10 times lower, as well as higher proliferation inhibition. Additionally, the differentiation of mouse embryonic stem cells (mESCs) toward endoderm was evaluated. The immobilized bFGF showed three times higher expression of endoderm differentiation genes in comparison to the control. In another study, the carboxylic groups of either BMP-2 or fibroblast growth factor-2 (FGF-2) were used to form covalent bonds with amine groups of chitosan films and, immobilize each GF using EDC/NHS chemistry for osteogenesis and wound healing (Budiraharjo et al., 2013). For BMP-2 and FGF-2, a loading efficiency of 64 and 50% was obtained, respectively. Regarding bioactivity, immobilized BMP-2 stimulated osteoblasts proliferation, differentiation and attachment of osteoblasts in a greater extent than the adsorbed GF. While the immobilized FGF-2 induced higher fibroblast attachment, proliferation, and collagen synthesis than the adsorbed GF.

##### Thiol coupling immobilization

One strategy in covalent immobilization uses thiol-reactive groups able to couple with thiol-containing molecules. Most of these thiol-reactive groups conjugate free thiol groups by one of two reactions: alkylation or disulfide exchange. Once initiated, these reactions generate either thioether bonds or disulfide bonds, respectively. In general, this technique involves the reaction of free thiols in the GF with thiol-reactive groups in the substrate surface. In GFs, free thiol groups exist in cysteine residues, but may also be introduced chemically or with recombinant technology (Place et al., 2012; Chen et al., 2014; Riahi et al., 2017). Functionalized GFs react with free thiols on the substrate surface, leading to their immobilization.

GFs have been prevalently immobilized through maleimides and, to a lesser extent, through vinyl sulfones, which in addition to reacting with thiol groups can also bind secondary targets such as amine and hydroxyl groups (Zisch et al., 2003; Ichinose et al., 2006; Rahman et al., 2010; He et al., 2011; Chen et al., 2014). Their selectivity depends on the pH of the reaction medium and the nucleophilic characteristic of target groups (Hermanson, 2008; Lopez-Jaramillo et al., 2012). Zisch et al. (2003) used polyethylene glycol (PEG) divinyl sulfone-functionalized hydrogels to immobilize VEGF, an Arginylglycylaspartic acid (RGD) peptide and a metalloproteinase (MMP) substrate peptide. Functionalized hydrogels induced angiogenesis, promoted human endothelial cells (HUVECs), adhesion and migration by cell-associated MMPs. Two VEGF variants were selectively immobilized, containing either no reaction site or two unpaired cysteines. Loading efficiency of the variants was surprisingly similar in both cases and close to 80%. This was suggested a result of a huge stoichiometric excess of PEG divinyl-sulfone, inducing the reaction of VEGF amine groups and PEG divinyl-sulfone, incorporating the GF into the hydrogels. Regarding bioactivity, both immobilized VEGF variants showed preserved functionality by promoting migration of HUVECs, inducing angiogenesis and vascularized tissue formation.

VEGF has also been immobilized onto functionalized agarose gels to control blood progenitor cell generation from mESCs aggregates (Rahman et al., 2010). To facilitated binding, VEGF was modified with maleimide reactive groups. To test the efficiency of immobilized VEGF, mESCs aggregates were encapsulated within VEGF immobilized agarose and cultured for 7 days as free-floating aggregates to assess the efficiency of immobilized VEGF in generating the desired phenotype. Immobilized VEGF was 75 times more efficient in inducing of mesodermal markers, brachyury and VEGF receptor 2, than its soluble counterpart by day 4. After 7 days, CD34^+^ and CD41^+^ expression, and generation of blood colony forming cells were 108 and 23 times higher. These results suggest that immobilizing GFs in a cell-hydrogel culture system is effective to enhance blood cell production, although large scale implementations remain to be tested.

Other thiol reactive groups, such as allyl ethers and norbornenes, may also be used for immobilizing GFs onto surfaces, as demonstrated with other proteins and peptides (Wittrock et al., 2007; Van Hove et al., 2014). Nevertheless, the selection of a specific reaction should be done on the basis of reaction conditions compatibility with GF functionality. For instance, the presence of by-products, required pH conditions, the use of organometallic catalysts and stability of the linkages formed should be taken into account (Lopez-Jaramillo et al., 2012).

##### Light-induced immobilization

Light can induce covalent immobilization of GFs to a substrate. This is based on the use of photoreactive groups, which are converted into highly reactive species covalently binding target molecules upon light exposure (Hermanson, 2008; Kawamoto et al., 2018). Usually, GFs are conjugated to a photoreactive group, then attached to the substrate surface upon exposure to a specific wavelength. Alternatively, substrate surfaces may be functionalized with photoreactive groups, which in turn react with GFs for immobilizing them into the surface. Light-induced immobilization facilitates spatial and temporal control over the immobilization process since the reaction is limited by light exposure parameters. However, light exposure may alter GF bioactivity due to stereochemical alteration, conformational changes, aggregation, and fragmentation which are protein specific (Pattison et al., 2012). These drawbacks may be avoided by reducing exposure time and using long-wavelength UV light (Masters, 2011).

Several photoreactive groups are available for functionalizing either GFs or substrate surfaces, including phenyl azide, benzophenone, anthraquinone, diazo compounds, and diazirine derivatives. Of these groups, phenyl azide has an important advantage: a low energy of activation, allowing short light exposure at higher-energy UV wavelengths, which avoids potential damage of photosensitive biomolecules and cells (Hermanson, 2008). When exposed to UV light, phenyl azide groups form nitrene groups, that undergo addition reactions with double bonds, insertion into carbon-hydrogen and nitrogen-hydrogen sites or subsequent ring expansion with amine groups and aliphatic compounds (Kawamoto et al., 2018). As an example of GF attaching via phenyl azide, Stefonek-Puccinelli and Masters (2008) conjugated EGF and IGF-1 to a crosslinker containing a phenyl azide group for immobilizing these GFs to polystyrene plates upon UV radiation (Stefonek-Puccinelli and Masters, 2008). Results showed that when GFs were immobilized individually, they stimulated keratinocyte migration in a greater extent than unmodified polystyrene plates. Interestingly, when both GFs were co-immobilized in the plates, they enhanced migration beyond levels achieved using individually immobilized GFs.

Another photoreactive group efficiently used for GF immobilization is benzophenone. In this approach, surfaces are conjugated with benzophenone, that upon UV light excitation forms a transient diradical that reacts with carbon-hydrogen sites from the GF to form a carbon-carbon covalent bond (Gomez et al., 2007; Hermanson, 2008; Martin et al., 2011). Unlike phenyl azide, unreacted benzophenone can be re-induced to an active state with subsequent UV exposure (Hermanson, 2008). Banks et al. (2014) conjugated photoreactive benzophenone to collagen-glycosaminoglycan (CG) scaffolds for immobilizing BMP-2 and platelet-derived growth factor-BB (PDGF-BB) either individually or together onto the same surface. Results indicated that the metabolic activity and proliferation of adipose-derived mesenchymal stem cells (ASCs) was impacted by immobilized PDGF-BB but not by BMP-2. Moreover, collagen 1 gene expression was downregulated with immobilized PDGF-BB, however it was strongly increased with the presence of BMP-2 alone or with PDGF-BB.

A commonly used photoreactive group for polymer covalent crosslinking is acrylate. The crosslinking of acrylate containing polymers is initiated by the generation of radicals usually formed from the photo-cleavage of initiator molecules. These radicals react with the unreacted carbon-carbon double bonds of the acrylated biomaterial resulting in covalently crosslinked polyacrylate chains (Bowman and Kloxin, 2008; Lin and Anseth, 2009). The same acrylate groups used for polymers photo-crosslinking are also potential sites for covalently immobilizing GFs as demonstrated with stem cell factor (SCF), basic fibroblastic growth factor (bFGF), VEGF and PDGF-BB (DeLong et al., 2005; Leslie-Barbick et al., 2009; Saik et al., 2011; Mahadik et al., 2015). In this method, the primary amine group of the GF is usually attached to a linker (typically PEG) which contains an acrylate group that in turn reacts with an acrylated substrate for conjugation of the GF.

Another common method for polymer crosslinking, specifically collagen, involves using riboflavin as a photoreactive group. Riboflavin (vitamin B2) is mainly used in ophthalmic and tissue engineering applications to enhance corneal strength and tailor mechanical properties of collagen constructs (Tirella et al., 2012; Rich et al., 2014; Hsu and Sugar, 2017). Recently, it has also been used for crosslinking GFs, such as EGF, bFGF, and TGF-β1, onto collagen-based biomaterials (Bertolo et al., 2015; Fernandes-Cunha et al., 2017). During exposure to UV light, oxygen species are released from the carboxylic groups of riboflavin, leading to the generation of light-activated riboflavin and single reactive oxygens. These highly reactive molecules then induce the formation of covalent bonds by reacting with the amino acids from the GF and collagen (Rich et al., 2014; Hsu and Sugar, 2017). It has been suggested that possible vulnerable amino acids to photochemical crosslinking using riboflavin include tyrosine, histidine, cysteine and methionine (Rich et al., 2014). Fernandes-Cunha et al. (2017) demonstrated that when EGF is crosslinked to collagen surfaces using riboflavin and blue light exposure, the histidine residues of this GF are engaged in the immobilization process. The photo-immobilized EGF maintained its bioactivity by enhancing the proliferation and spreading of corneal epithelial cells (CECs). Additionally, modified surfaces resulted cytocompatible and the photo-crosslinking reaction was not harmful to cells by preserving viability at values near 100%.

A primary advantage of light-induced immobilization over other covalent methods is that allows spatial and temporal control of the immobilization process. This characteristic has been exploited to create patterns of immobilized GFs onto two dimension surfaces and three dimension scaffolds by using UV light and photomasks (Stefonek-Puccinelli and Masters, 2008; Saik et al., 2011; Alsop et al., 2014; Banks et al., 2014). These patterns may provide a specialized effect of GFs in cellular functioning, since *in vivo*, soluble and ECM-bound biomolecules exist in gradient patterns that guide growth, migration, and differentiation of cells in a wide variety of tissues (Keenan and Folch, 2007). For example, Alsop et al. (2014) functionalized CG scaffolds with benzophenone to immobilize VEGF in spatially defined patterns, and evaluate their effect on HUVECs morphology by fluorescent staining. The scaffolds with patterned VEGF into geometric designs revealed morphological features of activated HUVECs such as branching, elongation, and increased cell-cell contact. Whereas, unmodified scaffolds displayed clumped HUVECs that didn't exhibit an activated morphology. These results indicate that GF patterns may be created by light-induced immobilization for directing cells bioactivity within a biomaterial.

##### Mussel-based immobilization

Mussel-based immobilization is inspired by the ability of marine mussels to attach to wet surfaces by secreting adhesive proteins rich in 3,4-dihydroxyphenylalanine (Dopa) and amine groups (Lee et al., 2007). The catechol side chain in Dopa confers these proteins the ability to bind various types of surface substrates and solidify *in situ* (Kord Forooshani and Lee, 2017). Dopamine, a small molecule containing both catechol and amine groups, is capable of immobilizing biomolecules, such as GFs, to a wide variety of organic and inorganic materials. Under alkaline conditions, dopamine polymerizes and forms adhesive polydopamine films that react with amine and thiol groups via Michael addition or Schiff base reactions (Lee et al., 2007). Polydopamine immobilizes GFs by reacting with amine and thiol groups of the GF and forming covalent bonds.

Yang et al. (2012) demonstrated that polydopamine mediated immobilization can be applied to several different GFs: VEGF, neural growth factor (NGF), bFGF and glial cell line-derived neurotrophic factor (GDNF). These GFs were individually immobilized onto polystyrene and PLGA surfaces, and their effect on human adipose stem cells (ADSCs), neural stem cells (NSCs), and HUVECs bioactivity was evaluated. The immobilization of bFGF and VEGF enhanced the proliferation of ADSCs cultured on PLGA surfaces but not on polystyrene surfaces. HUVECs proliferation was enhanced by bFGF and VEGF immobilized onto PLGA surfaces, ~2 and 3-fold, respectively. Whereas, bFGF and VEGF immobilization onto polystyrene surfaces, increased HUVECs proliferation around 0.5 and 0.7-fold, respectively. In the case of CDNF and NGF functionalized surfaces, differentiation and proliferation of NSCs was enhanced. This study demonstrates that mussel-based immobilization is a technique that allows functional culture of stem cells and primary cells. Other studies have also reported covalent immobilization of GFs onto dopamine treated surfaces for tissue engineering applications (Poh et al., 2010; Lai et al., 2011; Kang et al., 2012, 2013a). Since GFs and surfaces do not require complex modification procedures, mussel-based immobilization is considered simpler than other covalent immobilization techniques (Poh et al., 2010).

##### Other covalent methods

Several other chemistries have been used to immobilize GFs covalently onto various surfaces. Plasma treatment involves the introduction of functional groups to surfaces, which can be utilized in various chemistries, such as carbodiimide coupling, to immobilize GFs (Zhang et al., 2012). This particular technique has been used for the immobilization of bone morphogenetic protein-4 (BMP-4), BMP-2, bFGF, and FGF-2 (Puleo et al., 2002; Shen et al., 2008, 2009; Kokubu et al., 2009). Silane and imine coupling are other useful techniques for GF immobilization, in which the formed covalent bonds can be hydrolyzed under physiological conditions, allowing control on subsequent release of the GF (Cabanas-Danés et al., 2018).

#### Non-covalent Approaches for GF Tethering

A major advantage of using non-covalent immobilization approaches is that in most cases the GF is simply added to target substrates without the need of a prior step of GF modification. Also, the majority of these approaches allow oriented immobilization of the GF because it is possible to select the attachment site of the GF onto the surface. Whereas, in most of the covalent approaches, reactive groups are expected to interact with a specific functional group that may be present more than once in the GF, immobilizing it in a random orientation, which in consequence can affect bioactivity (Nakaji-Hirabayashi et al., 2007, 2008). Another attractive feature of non-covalent approaches is that most of them are reversible, allowing temporal control of the immobilized GF. An overview of non-covalent approaches is included in Figure 2.

##### ECM-based immobilization

The ECM is a highly dynamic and complex network composed of glycoproteins, proteoglycans, collagen, and glycosaminoglycans. ECM functions are related to cellular processes, serve as a reservoir of GFs, and modulate their bioavailability (Kim et al., 2011). Among the diverse GF-ECM interactions, the largest group involves GFs binding to heparin or heparan sulfate. GFs interact with heparin *in vivo* through specific binding domains, and electrostatic interactions, which take place between negatively charged carboxyl and sulfate groups from heparin and GFs positively charged amino acids (Taipale and Keski-Oja, 1997; Joung et al., 2008). In an attempt to mimic the natural biological environment of GFs, and thus, maintain signaling events that occur *in vivo*, various substrates surfaces have been functionalized with heparin or heparan-sulfate to immobilize a wide variety of GFs (Kato et al., 2007; Freudenberg et al., 2015; Jha et al., 2015; Kim et al., 2015, 2016a; Ma et al., 2015; Shin et al., 2015). Interestingly, Kim et al. (2016a) exploited the occurring electrostatic interactions between GFs and heparin to develop an electrochemically based GF release system that disrupts these interactions. This approach demonstrated effective control of bFGF release from heparinized titanium surfaces paired with stable bioactivity.

The attributes of heparin, such as the sulfation pattern and molecular weight, have relevant consequences in bioactivity, loading efficiency, and release of immobilized GFs. In this context, Jha et al. (2015) demonstrated that heparin molecular weight and concentration affected the loading and retention of TGF-β1 in hyaluronic acid-based matrices, primarily through greater affinity of TGF-β1 to high molecular weight heparin (Jha et al., 2015). In another study, the sulfation pattern of heparin modulated the release of VEGF from starPEG-heparin hydrogels and influenced VEGF pro-angiogenic action *in vitro* and *in vivo*. The desulfation of heparin resulted in higher release of VEGF, occasioning a higher angiogenic cell response *in vitro* (Freudenberg et al., 2015).

An alternative to substrate surface functionalization with heparin and heparan sulfate is to use other ECM molecules that also interact with GFs *in vivo*, such as collagen, fibrinogen, fibrin, betaglycan, and decorin (Kato et al., 2007; Macri et al., 2007; Schultz and Wysocki, 2009; Sawicka et al., 2015). For instance, Martino et al. (2011) generated a fibrin matrix functionalized with a fibronectin recombinant fragment displaying integrin and fibronectin binding domains to simultaneously control GF and integrin binding (Martino et al., 2011). In this approach, three GFs were immobilized individually: VEGF, PDGF-BB, and BMP-2. *In vitro* experiments showed that fibrin containing immobilized VEGF increased proliferation and migration of endothelial cells about 10% more compared to the soluble GF. Mesenchymal stem cells (MSCs) cultured on fibrin matrices functionalized with BMP-2 didn't had a significant effect on proliferation and migration. Whereas, MSCs cultured on PDGF-BB functionalized matrices showed a 3% increase in proliferation and migration compared to the soluble GF. Finally, fibrin matrices with immobilized PDGF-BB enhanced proliferation around 4%, and migration around 14%. Furthermore, the fibrin matrices with immobilized GFs enhanced the regenerative effects of GFs *in vivo* in a diabetic mouse model of chronic wounds and in a rat model with calvarial defects. These results suggest that immobilization methods inspired in ECM functional components are able to induce a proper cellular response, as well as improving cell bioactivity and tissue regeneration *in vivo* (Martino et al., 2011).

##### Peptide-based immobilization

Peptides are short oligomers of amino acids synthetized through well-established methods and used in many applications, including the attachment of biomolecules, such as enzymes, proteins, antibodies and GFs, to a wide variety of surfaces (Naffin et al., 2003; Jung et al., 2008; Wang et al., 2008; Lin et al., 2009; Fu et al., 2011; Cabanas-Danés et al., 2013; Crispim et al., 2017). For GF immobilization, surfaces are functionalized with specific binding peptides that interact with a pre-loaded GF through different possible modes, such as hydrophobic interactions, recognition of secondary structure motifs, and electrostatic interactions (Stanfield and Wilson, 1995; Wrighton et al., 1996; Fairbrother et al., 1998; Wang et al., 2008). These peptides are often identified by a screening of several sequences that display different properties and thus, bind to their target (in this case GFs) with varying affinity, specificity, and strength (ten Brummelhuis et al., 2017). Specific binding peptides inspired in naturally occurring binding domains may also be used for GF immobilization. It is important to mention that surface functionalization is not the only approach for peptide-based immobilization of GFs, fusion proteins of GFs with specific binding peptides may also be utilized for this purpose and will be described further on in the genetic fusion section.

As an example of peptide-based immobilization, Wang et al. (2008) attached VEGF to collagen scaffolds by using a modified collagen mimetic peptide (CMP) with multiple anionic charges at the N-terminus, designed to bind collagen by strand invasion, and simultaneously attract VEGF through charge-charge interactions. Since collagen binding occurs only by contact with melted, single-stranded CMP, two collagen treatments involving either a hot CMP solution (hCMP) or a CMP solution quenched from 80 to 25°C (qCMP) were evaluated. The loading efficiency of VEGF onto collagen scaffolds treated with hCMP and qCMP was approximately 32 and 20.5%, respectively. Moreover, the collagen scaffolds treated either with hCMP or qCMP after VEGF addition and HUVECs seeding, enhanced tube-like morphology in cells, and activated the integrin-link kinase angiogenic pathway, indicating a biologically relevant cell response to immobilized VEGF.

Besides surface functionalization with specific binding peptides, self-assemble peptide amphiphiles (PAs) have also been used for peptide-based GF immobilization (Hosseinkhani et al., 2006; Hsieh et al., 2006; Stupp et al., 2010). PAs are peptide-based molecules containing a hydrophilic tail and hydrophobic head composed of several amino acids, that mimic surfactant structures (Qiu et al., 2018). In order to confer biological activity to these peptides, they are conjugated to bioactive epitopes that are placed as the hydrophilic tail, since the hydrophobic head must be conserved to ensure the self-assembling process (Cui et al., 2010; Qiu et al., 2018). PAs self-assembly into various nanostructures in aqueous solutions at certain pH, ionic strength and temperature conditions, resulting in exposure of the bioactive epitopes on the nanostructure surface (Dehsorkhi et al., 2014). Depending on the desired biological response, different epitopes may be conjugated to PAs, particularly for GF immobilization, specific GF binding peptides may be used.

In a pioneering study, PA nanofibers that displayed a high density of TGF-β1 binding epitopes at the surface to immobilize TGF-β1 were employed for use in cartilage regeneration (Stupp et al., 2010). The PA nanofibers containing TGF-β1 binding epitopes (TGFBPA) were mixed with non-bioactive PAs that acted as a filler to ensure adequate epitope binding and GF display. GF release studies showed a 3-fold slower TGF-β1 release after 72 h when immobilized in TGFBPA scaffolds. *In vitro* experiments demonstrated that TGFBPA scaffolds were able to support MSCs viability and chondrogenic differentiation as well as leading to an increase in gene expression of cartilage markers. The *in vivo* potential of PA scaffolds to promote cartilage regeneration in the presence of bone marrow MSCs was evaluated in adult rabbits with microfractures in the trochlea. Macroscopic and histological evaluation of the defects revealed that in contrast with TGF-β1 alone and filler scaffolds with TGF-β1, TGFBPA scaffolds (with and without TGF-β1) enhanced tissue regeneration. In terms of a modified version of the O'Driscoll 24-point scoring system, TGFBPA scaffolds with and without TGF-β1 had approximately a 1.5-fold higher score, indicating higher quality of the new tissue. Bioactivity showed in TGFBPA scaffolds without exogenous GF was explained as a result of binding events of endogenous TGF-β1 to epitopes present in the scaffold.

##### Coiled-coil interactions

The coiled-coil is an oligomerization domain found in ~3–5% of all proteins, involved in a wide range of biological functions (Mason and Arndt, 2004; Truebestein and Leonard, 2016). Examples of coiled-coil-containing proteins include extracellular and motor proteins, such as kinesin, myosin, keratin, and fibrin, as well as transcription factors, such as Jun and Fos (Mason and Arndt, 2004; Goktas et al., 2018). Usually, coiled-coil consists of two to five α-helices, parallel or antiparallel, wound into super-helical structures. The structure of coiled-coil proteins is characterized by a seven amino acid (heptad) repeat, denoted *abcdefg*, that directs folding and dimerization of the helices (Goktas et al., 2018). The positions a and d are hydrophobic amino acids that form the hydrophobic core of the coiled-coil and are packed in a knobs-into-holes arrangement, in which the residue from one helix (knob) packs into a space surrounded by four residues (hole) of the facing helix (Lupas and Gruber, 2005). The amino acids located at positions e and g are oppositely charged polar residues that flank the hydrophobic core and contribute to the coiled-coil structure stability by forming intra-strand salt bridges. Finally, b, c and f are more variable residues, with great relevance for stability (Goktas et al., 2018).

Due to their highly specific and stable interaction, coiled-coil structures have been used in various biomedical and biotechnological applications, including GF immobilization (Boucher et al., 2008, 2010; Apostolovic et al., 2010; Murschel et al., 2013; Assal et al., 2015; Noel et al., 2016; Riahi et al., 2017). The target GF is expressed as a fusion protein with one coiled-coil strand, while the substrate surface is functionalized with the complementary coiled-coil strand. Although naturally occurring coiled-coils may be used for GF immobilization, most studies appeal to *de novo* coiled-coils because their structure and properties, such as stability, degree of oligomerization, helix orientation, sensitiveness to pH and temperature, and self-assembly, can be controlled by protein/peptide engineering (Apostolovic et al., 2010). For example, De Crescenzo et al. (2003) designed a *de novo* coiled-coil system conformed by two peptides, designated Ecoil and Kcoil, with varying affinity and stability according to the number of heptads in the Ecoil and Kcoil. A five long heptad repeat in both Ecoil and Kcoil demonstrated high affinity and relative rapid association. This coiled-coil pair was used to immobilize EGF in an oriented manner on polyethylene terephthalate (PET) films and promote CECs response (Boucher et al., 2010). The Ecoil peptide was fused to the N-terminus of EGF while the Kcoil peptide was grafted in PET films. The loading efficiency of Ecoil-EGF onto Kcoil-functionalized surfaces was ~88%. When compared to adsorbed and soluble EGF, the immobilized GF enhanced CECs adhesion, proliferation and spreading.

##### Genetic fusion

Genetic fusion is another useful technique for GF non-covalent immobilization. It uses recombinant technology to generate fusion proteins of the GF with specific components, such as affinity tags and specific binding peptides (Arnau et al., 2006). Affinity tags show particular affinity to chemical or biological ligands, which are linked on the substrate surface to enable immobilization. As listed in Table 1, a wide variety of affinity tags and their ligands have been used for tethering GFs onto natural and artificial substrates, including: avidin and biotin; antibodies and antigens; histidine and metals; glutathione-s-transferase (GST) and glutathione (GTH); and maltose-binding protein and maltose (Kato et al., 2005; Ogiwara et al., 2005; Han et al., 2009; Kolodziej et al., 2011; Worrallo et al., 2017). In most cases, the interaction between the affinity tag and the ligand is reversible through the addition of competitive agents. Although histidine and biotin belong to the most commonly used affinity tags for immobilization purposes, they will not be described in this section, as their special case is described further on. Of note, GFs can also be immobilized through affinity interactions without the necessity to fuse the GF to a tag. In a pioneering study, an affinity tag-free immobilization strategy was established by exploiting the native sugar lectin-interaction between glycosylated recombinant BMP-2 and concanavalin A (Wang et al., 2020).

**Table 1:** Genetic engineered binding GFs to natural and artificial substrates.

	**Substrate**	**Fused protein**	**GF**	**Reference**
Natural	Collagen	IgG	EGF	Ogiwara et al., 2005
	GSH- functionalized nanopatterns	Glutathione-s-transferase	FGF2	Kolodziej et al., 2011
	Gelatin and Fibrillar collagen sponges	Fibronectin collagen-binding domain	EGF	Ishikawa et al., 2001
	Beta Tricalcium Phosphate (βTCP)	βTCP-binding peptide	EGF	Alvarez et al., 2015
	Collagen	Collagen binding domain	HGF	Kitajima et al., 2007
	Cellulose	Cellulose-binding domain	SCF	Doheny et al., 1999
	Silk coated surfaces	Spider silk protein	bFGF	Thatikonda et al., 2018
	Fibrin	Transglutaminase activity of factor XIIIa	?-NGF	Sakiyama-Elbert et al., 2001
	Fibrin	Transglutaminase activity of factor XIIIa and plasmin substrate	BMP-2	Schmoekel et al., 2004
	Fibrin	Transglutaminase activity of factor XIIIa	VEGF	Zisch et al., 2001
Artificial	Gold-coated glass plate	Hexahistidine residues	EGF	Kato et al., 2005
	Polystyrene surfaces	Maltose-binding protein	VEGF	Han et al., 2009
	Titanium surfaces	Titanium-binding peptides	hEGF	Tada et al., 2014
	Hydroxypatite	Statherin active site	EGF	Kang et al., 2013b
	Titanium surfaces	Statherin active site	EGF	Kang et al., 2013b
	Hydroxypatite	Diphosporylated serines from statherin	hBMP4	Sakuragi et al., 2011

Kolodziej et al. (2011) exploited the reversibility of the affinity linkage between GST and GTH to reutilize surfaces where FGF-2 had been previously immobilized. First, a fusion protein of FGF-2 and GST (FGF-2-GST) was immobilized onto GTH functionalized PEG hydrogel surfaces. Then, FGF-2-GST was released from the hydrogel surfaces upon free GTH addition. The surfaces were completely free from FGF-2-GST and used successfully to bind a second FGF-2-GST. Therefore, GF immobilization through reversible affinity tags may open the possibility for performing iterative immobilization on a single substrate surface, contributing to further decrease of cell culture costs. However, to elucidate the potential application of this approach, the effect of iteratively immobilized surfaces on bioactivity must be investigated.

In a previous section, GF immobilization through peptides was described, however focus was put on surface functionalization with specific binding peptides and not on genetic fusion. In the latter approach, peptide sequences that have an affinity to certain materials or proteins are selected and used for fusion with GFs through recombinant technology. A wide variety of peptides that bind to different substrates including collagen, cellulose, hydroxyapatite, titanium, polystyrene, and beta-tri calcium phosphate, have been used to immobilize GFs (Doheny et al., 1999; Ishikawa et al., 2001; Kitajima et al., 2007; Kang et al., 2013b; Tada et al., 2014; Alvarez et al., 2015; Thatikonda et al., 2018). These peptides are derived from combinatorial screens of peptide libraries or based on naturally occurring binding domains. Although a GF may exhibit affinity to a substrate, avidity of the binding may improve by fusing additional binding domains to the GF. For example, Kitajima et al. (2007) improved the binding affinity of native hepatocyte growth factor (HGF) to collagen 16 times by fusing HGF to a polypeptide derived from the fibrin collagen binding domain (CBD) (Kitajima et al., 2007). In comparison to soluble HGF, the immobilized CBD-HGF promoted HUVECs cell growth for 4 extra days longer, overall yielding 5 times more cells over 10 days. Although recombinant technology is widely used for conjugating GFs to peptides, peptide ligation can also be used for this purpose. To our knowledge, this technology has been used in only example reported to date, in which human bone morphogenetic protein 4 (hBMP4) was attached to hydroxyapatite beads (Sakuragi et al., 2011).

Fusion proteins containing GFs may also include domains to provide additional functionalities. For instance, a fusion protein made of neural growth factor-β (NGF-β) with two functional domains: a factor XIIIa transglutaminase domain (TG) as incorporation site to fibrin matrices, and plasmin substrate domain (P) which provided a cleavage site for the local release of the iGF under cell-activated plasmin (Sakiyama-Elbert et al., 2001). The surface functionalization of fibrin matrices with this fusion protein (TG-P-NGF-β) enhanced neurite extension of neural crest-derived PC12 cells by 50% more compared to native NGF-β. The high bioactivity of TG-P-NGF-β suggested that the iGF was effectively released from the matrices in an active form. In this setting, TG mediated covalent attachment of the fusion protein to the matrices. After thrombin-mediated activation, TG catalyzes the formation of covalent bonds between glutamine and lysine residues in fibrin chains (Corbett et al., 1997). The TG domain has also been used for the covalent immobilization of VEGF and BMP-2 on fibrin matrices (Zisch et al., 2001; Schmoekel et al., 2004).

##### Biotin-streptavidin interactions

One of the strongest non-covalent interactions known involves the binding of biotin to avidin or streptavidin. Both of these proteins contain four subunits able to bind biotin with great affinity and specificity (Hermanson, 2008). However, avidin is positively charged and may generate non-specific interactions, while the neutral variant streptavidin does not present this characteristic and may be the preferred alternative (Nguyen et al., 2012). Usually, the immobilized surface is functionalized with either avidin or streptavidin, while biotin is coupled to the molecule of interest. In some instances, it is necessary to test GF immobilization efficiency depending on either the biotin-GF binding or the biotin-streptavidin binding occurring first (Moore et al., 2017). In most approaches, GF immobilization by biotin-streptavidin interactions involves the use of tetrameric streptavidin, which makes the biotin-avidin/streptavidin interaction non-reversible. As an alternative, monomeric streptavidin may be used to produce reversible binding, allowing temporal control of the immobilized GF. Monomeric streptavidin has a reduced affinity for biotin compared to tetrameric streptavidin due to its considerably smaller dissociation constant (Wu et al., 2009). Other approaches to reverse biotin-streptavidin interactions include incubation in high temperature aqueous solutions, development of tetrameric streptavidin mutein with lower affinity for biotin, design of biotin analogs with lower affinity for streptavidin (Holmberg et al., 2005; Ying and Branchaud, 2011; O'Sullivan et al., 2012).

GFs are usually biotinylated using biotin derivatives containing reactive groups that specific for coupling to a particular functional group on the GF. However, the biotin derivative must be carefully selected to ensure preserved bioactivity of the GF (Hermanson, 2008). Common biotin derivatives for GF modification are the amine-reactive, such as sulfo-NHS-biotin (Shahal et al., 2012; Kim et al., 2016a; Worrallo et al., 2017). The NHS ester of this compound reacts with the GF primary amines to form an amide bond and thus, couple with biotin. Some biotinylating reagents may also contain spacer groups, such as the NHS-PEG-biotin, which may improve the binding potential toward avidin or streptavidin by reducing steric hindrance, increase the solubility of the reagent, and increase control over the steric presentation of GFs to target receptors (Hermanson, 2008; Cipolla et al., 2013; Worrallo et al., 2017). It should be highlighted that the use of a spacer group is not limited for biotin-streptavidin based approaches. A spacer group may be attached to a wide variety of affinity ligands or functional groups, such as maleimide, vinyl sulfones and carboxylic acids. GF biotinylation can be achieved using recombinant technology, with GFs expressed as fusion proteins with a biotin label incorporated at the N-terminus (Leipzig et al., 2011; Li et al., 2014).

Recently, Worrallo et al. (2017) developed a potential immobilization strategy for controlling GFs presentation in cell suspension culture platforms (Worrallo et al., 2017). GFs were biotinylated via reaction of NHS on NHS-PEG-biotin with primary amines on lysine residues of GM-CSF, SCF and hematopoietic growth factor thrombopoietin (TPO). Biotinylated GFs were attached to magnetic streptavidin-coated particles. The magnetic properties of these particles allowed temporal control over GFs presentation. Immobilized SCF, GM-CSF, and TPO maintained bioactivity in GF dependent cell lines M-07e and TF-1. Using immobilized GM-CSF (iGM-CSF) permitted a 98.5% decrease in the use of the GF, compared to soluble GM-CSF (sGM-CSF) used over 192 h of culture. Interestingly, iGM-CSF retained functionality under agitation in a micro-scale stirred tank bioreactor and after short exposure, higher cell growth was obtained relative to sGM-CSF. Taken together, these results demonstrate that this biotin-streptavidin based approach is promising for reducing overall manufacturing costs of GF dependent cell culture systems, such as those being developed for cell therapies, by diminishing GF quantities required to induce a maximum cellular response. Magnetic recollection of iGF post-culture may also open the possibility for GFs recycling in the allogenic setting.

##### Chelator histidine tag interactions

A chelator-based immobilization technique, commonly used for protein purification in chromatographic processes, uses a histidine tag in combination with metal ions immobilized in chelators, such as iminodiacetic acid (IDA) and nitrilotriacetic acid (NTA). Histidine forms coordination bonds with several metal ions, such as Cu^2+^, Ni^2+^, and Zn^2+^ (Kimple et al., 2013). Based on the chelator and metal ion used, a number of coordination sites are available in the metal ion for interaction with the histidine tag. For example, when Cu^2+^ is chelated by NTA, it has one free site for interaction with histidine residues, whereas Ni^2+^ has three free sites (Hochuli et al., 1987). To immobilize a GF, a histidine tag is placed on either the N-terminus or C-terminus of the GF using recombinant technology, while the substrate surface is functionalized with a chelator and metal ion. The number of histidine residues in histidine tags may vary, however six histidine tags are generally recognized as adequate for yielding high affinity interactions with metal ions. This type of immobilization can be reversed by the addition of metal-chelating agents, such as ethylenediaminetetraacetic acid (EDTA), or competitive agents, such as imidazole (Bornhorst and Falke, 2000).

For example, Kato et al. (2005) immobilized EGF onto culture plates through the linking of a hexahistidine tag to its C-terminus, which binds to metal ions chelated to self-assembled monolayers (SAM). Surfaces linked to immobilized EGF (iEGF), allowed cell adhesion and proliferation. In comparison, surfaces with iEGF using carbodiimide coupling, exhibited few aggregated cells. When EGF was immobilized by carbodiimide coupling, cell adhesion was hardly observed, while cells cultured onto EGF chelated surfaces could attach and proliferate (Nakaji-Hirabayashi et al., 2007). This is due to the random orientation of the GF in surfaces with EDC/NHS chemistry mediated immobilization. The intact EGF structure after immobilization by chelation as well as firm immobilization onto the surface was demonstrated. Furthermore, EGF immobilization by EDC/NHS chemistry provoked GF denaturation (Nakaji-Hirabayashi et al., 2008).

## Case Study: Stem Cell Factor Immobilization

SCF exists both as a transmembrane and a soluble protein. It is produced by endothelial cells in the hematopoietic stem cell (HSC) niche, and binds to its receptor, CD117 also known as c-kit, on a variety of early hematopoietic cells, promoting their maintenance and proliferation (McNiece and Briddell, 1995). Due to its effect on HSC, soluble SCF (sSCF) has been used to produce a wide variety of therapies based on blood-lineage cells (Timmins et al., 2009; Zonari et al., 2017). In the context of cell therapy manufacture, immobilization could reduce consumable use and labor costs, which could in turn reduce the overall production cost and make cell therapies more accessible. In this context, investigating efficient strategies for SCF immobilization is highly relevant. Several research articles describe immobilizing SCF, and report feasibility and performance on specific cell line expansion processes. However, the economic impact of using this approach for cell therapy production remains to be determined experimentally.

As methods used to immobilize GFs are varied, several aspects must be considered when selecting the appropriate technique for a specific GF. SCF has several functional groups, which can be exploited for attachment to a variety of surfaces. For instance, 22 lysine residues from SCF can be engaged for using covalent methods using amine groups for immobilization, such as carbodiimide coupling and mussel-based immobilization (Jiang, 2000). SCF also contains 5 cysteine residues that can exploited for immobilization based on the use of thiol groups, such as thiol coupling, mussel-based, and light-induced immobilization (Jiang, 2000). Since PEG linkers commonly contain a reactive functional group, a PEG linker may be used to immobilize SCF to surfaces in both covalent and non-covalent methods. For example, PEG-NHS reacts with amine groups in lysine residues from SCF. Paradoxically, as SCF contains more than one amine group, attachment of the linker is not specific to a single site on SCF, which may affect the SCF active site and bioactivity. To avoid this, PEG linked to reactive groups of increased specificity can be selected, such as PEG-CHO and PEG-MAL, both used for specific targeting of N-terminal residues (Agusti et al., 2016).

A pioneering study reporting immobilization of SCF was done by Doheny et al. (1999), in which SCF was fused to a cellulose binding domain using recombinant technology to allow adsorption in a cellulose matrix. Immobilized SCF (iSCF) yielded a 5- to 7-fold increase in cell expansion compared to using similar concentrations of sSCF when culturing murine and human suspension bone marrow cell lines (Doheny et al., 1999). In a more recent study, Cuchiara et al. (2013) covalently immobilized SCF to PEG hydrogels to culture primary hematopoietic cell populations. A loading efficiency of 80% was achieved and the capacity of iSCF to induce proliferation of murine HSCs remained similar to that of sSCF. However, HSCs spreading was decreased, and cells exhibited a more rounded morphology when iSCF was used compared to sSCF, which is more in accordance to their native physiological state inside the bone marrow (Cuchiara et al., 2013).

Mahadik et al. (2015) immobilized murine SCF to methalcrylamide gelatin (GelMA) hydrogels using PEG-NHS as a linker between the functionalized matrix and SCF. Both sSCF and covalently attached SCF induced similar levels of proliferation in murine HSCs. Retention profiles were performed for covalently attached hydrogels and hydrogels with adsorbed SCF. After 7 days, covalently attached hydrogels retained 80% of the initial concentration of SCF, whereas adsorbed hydrogels only retained 40%. In addition, covalently attached hydrogels induced higher proliferation levels than adsorbed hydrogels. Therefore in the context of HSC *ex vivo* proliferation, SCF covalent attachment may promote improved cell proliferation while stabilizing the GF (Mahadik et al., 2015). In a different study, a non-covalent immobilization approach was used to attach SCF onto magnetic beads using PEGylation of SCF and biotin-streptavidin interactions (Worrallo et al., 2017). Results suggest immobilization-induced stabilization of SCF and possible iSCF dose reduction in cell culture. The SCF-dependent human cell line M-07e exhibited a 65% decrease in viability less when using iSCF at a dose corresponding to only 8% of the sSCF dose. However, the cell response to increasing iSCF concentrations was curvilinear and did not reach sSCF response. Interestingly, SCF could be co-immobilized with additional relevant GF such as TPO y GM-CSF, on the surface of magnetic beads at controlled concentrations. This strategy is particular useful if co-signaling is required in specific cell production processes and is currently used to activate T cells with anti-CD3/anti-CD28 for the production of CAR T cell therapies.

Taken together, these results demonstrate differential effects of iSCF on cell proliferation and phenotype depending on the nature of target cell and the immobilization method employed. Overall, iSCF improves cell culture processes, and current results evidence the need for further research into exploiting potential additional benefits, for example the temporal and spatial control over iSCF presentation to cells.

## Concluding Remarks

Cell therapies promise innovative treatments for a variety of life-threatening conditions and the number of clinical trials of cell-based therapies is rapidly increasing. A downfall of these treatments is their high cost of production, which translates into a high selling price hampering their widespread use. Acquisition of materials, especially GFs, is a major contributor to the overall cost of production. GF immobilization has emerged as a strategy to optimize a rational GF usage in cell culture, contributing to reduction of costs.

A wide variety of techniques are available for immobilizing GFs to different biomaterials. Covalent immobilization techniques usually allow a slower release profile of the GF compared to physical approaches. However, they may engage functional groups required for induction of a cellular response. Of note, if functional groups used for immobilization are present more than once in the GF, immobilization may occur in a random orientation for example, which can affect bioactivity. On the other hand, non-covalent immobilization techniques allow a more specific immobilization because it is possible to select the precise attachment site within the GF sequence in most cases. Furthermore, in most of non-covalent techniques, temporal control is easily achieved, since reversible tags can be used. Physical immobilization techniques typically result in a faster release of the GF, resulting in poor spatial and temporal control over GF presentation to cells, but they do not compromise the availability of functional groups.

In many cases, the techniques presented in this review were specifically applied to immobilize one specific GF. However, to replicate a native cell niche and induce a physiological cellular response, co-signaling with additional GFs is often required. Therefore, immobilization of multiple GFs onto a single surface is highly desirable and represents a relevant field of investigation. Additionally, the combination of multiple techniques may be exploited to tailor the release of GFs and have increased control over their steric presentation to cells.

The impact of GF immobilization on reducing the overall manufacturing cost of cell therapy vary with a number of parameters including the cost of the GF itself, and its specific half-life and loading efficiency. The selected immobilization method also dictates the immobilization support to be used, impacting the cost. Some methods are more sophisticated than others, which demand and increased initial inversion to perform the immobilization. Physical immobilization methods are generally the most cost-effective but entail disadvantages such as lack of control-over-release. Methods involving genetic fusion of an adapter to the GF are more expensive because a recombinant fusion protein must be designed and produced. The cost of the substrate or immobilization surface also factors into final immobilization cost. For instance, magnetic beads are more expensive than a matrix scaffold. In addition to cost, efficiency and specific biological features induced by each of the immobilization methods should be considered when selecting the most appropriate technique to optimize GF usage aiming for overall cost reduction without decreasing cell response. An economical evaluation is critical to evaluate the real impact of GF immobilization in cell manufacturing costs.

It is clear that GF immobilization is a strategy that addresses many of the issues related to the use of soluble GFs. However, most of the immobilization techniques have focused on immobilizing GFs for tissue engineering applications involving cells growing in adherence. The few studies performed on suspended-cells culture systems show promising results by reducing significantly the total amount of GFs required. Further research is needed to elucidate the full potential of GF immobilization to optimize cell production processes, including potential for re-use.

## Author Contributions

DE-O wrote sections methods for GFs immobilization, physical immobilization of GFs, case study: stem cell factor immobilization, and concluding remarks and developed Figure 1. PR-O wrote sections introduction, cost of cell therapy development and manufacturing: flipside of the coin, case study: stem cell factor immobilization, and concluding remarks. MB and KM-D provided initial context and structure for this manuscript, wrote and edited all content. All authors provided critical feedback and approved the final version of the manuscript.

## Conflict of Interest

The authors declare that the research was conducted in the absence of any commercial or financial relationships that could be construed as a potential conflict of interest.
