# Corrigendum: Immobilization of Growth Factors for Cell Therapy Manufacturing

**DOI:** 10.3389/fbioe.2020.00821

**Published:** 2020-07-31

**Authors:** Daniela Enriquez-Ochoa, Pedro Robles-Ovalle, Karla Mayolo-Deloisa, Marion E. G. Brunck

**Affiliations:** Tecnologico de Monterrey, School of Engineering and Science, FEMSA Biotechnology Center, Monterrey, Mexico

**Keywords:** cell therapy, growth factor, immobilization, stem cell factor, cost-of-goods, cell product manufacturing

In the original article, there was a mistake in Figures 1, 2 as published. The order of the figures was reversed while the legends of these figures were correct.

**Figure 1:**
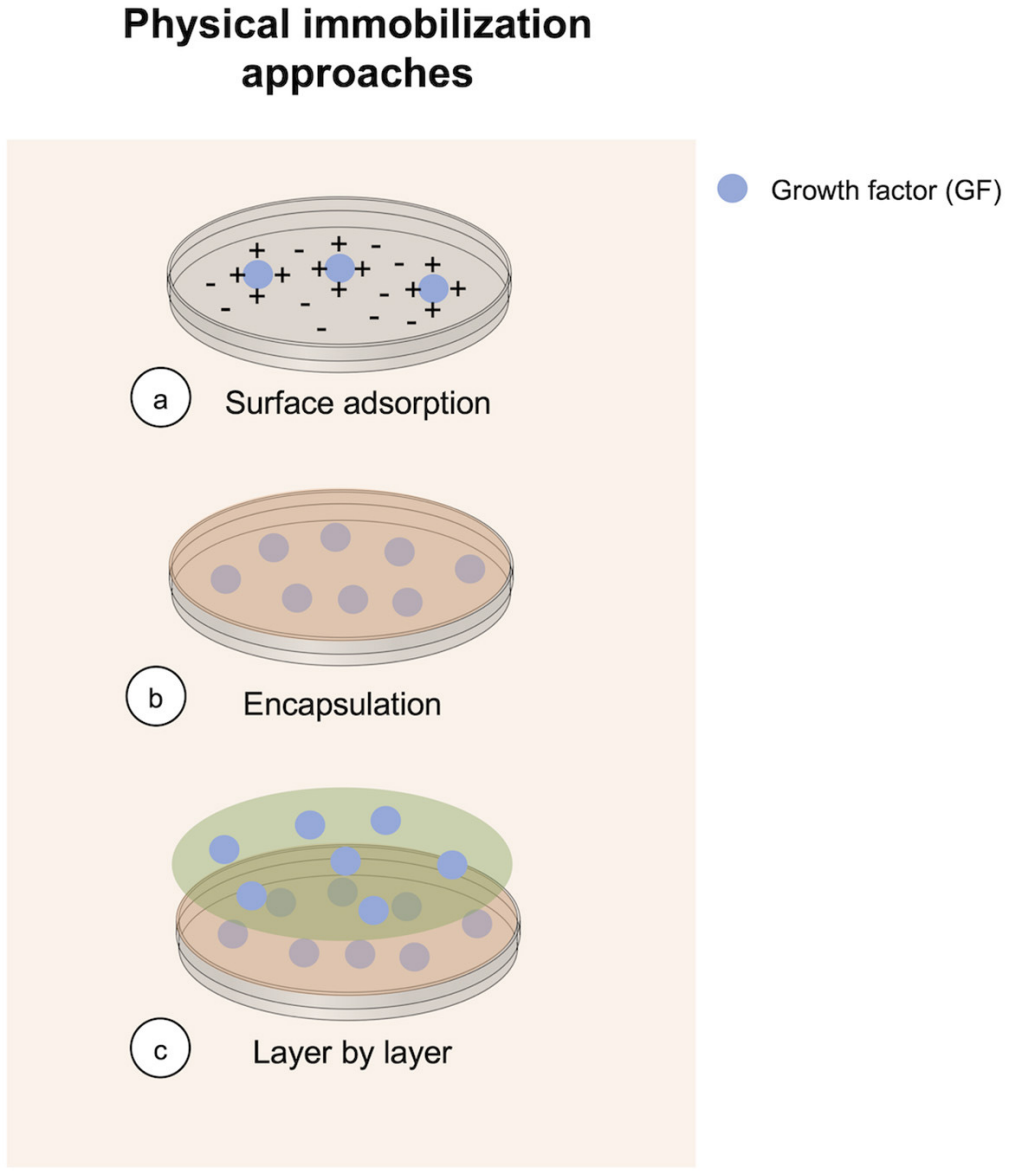
Overview of methods currently in use to perform physical GF immobilization.

**Figure 2:**
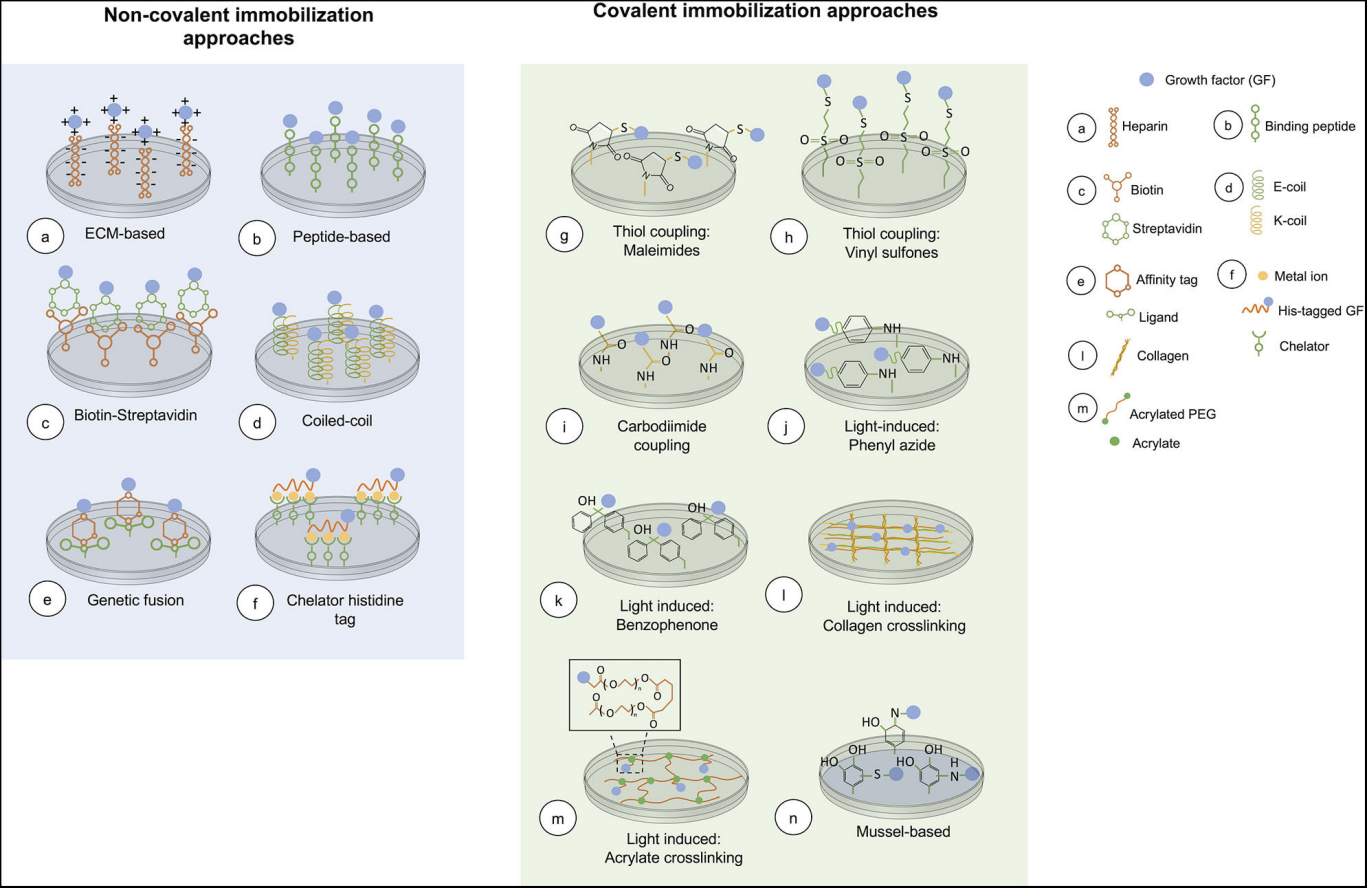
Overview of current chemical immobilization strategies. In covalent methods, acrylate crosslinking is representative of the conjugation of an acryloyl GF and PEG-diacrylate matrix. While, mussel-based immobilization shows the interaction between the GF and polydopamine coated on the surface.

The corrected Figures 1, 2 appear below.

The authors apologize for this error and state that this does not change the scientific conclusions of the article in any way. The original article has been updated.

